# Flapless implant surgery: A review of the literature and 3 case reports

**DOI:** 10.4317/jced.51985

**Published:** 2015-02-01

**Authors:** Manuel-Maria Romero-Ruiz, Regina Mosquera-Perez, Jose-Luis Gutierrez-Perez, Daniel Torres-Lagares

**Affiliations:** 1 Professor of the Oral Surgery Master – University of Seville; 2 Oral Surgery Master – University of Seville; 3 Head of the Oral Surgery Master – University of Seville

## Abstract

Since the 1970s, modern Implantology is based on a concept of surgery with flap elevation. Gradually, several clinical trials demonstrated that a mid-crestal incision gives similar success rates compared to those obtained using the classical protocol. However, over the past decade in medicine it has been established the concept of minimally invasive surgery, consisting in taking advantage of advancements experienced in diagnostic techniques and specific surgical instruments, to perform surgical procedures infringing as less damage as possible to the patient The present work aims to produce a thorough review of the literature published on the field of Implantology with flapless surgery, to determine the current scientific evidence of the technique, along with illustrating the results with different clinical cases. After presenting the clinical cases, and the review of literature, we can say that flapless surgeries should be restricted to well-selected cases in which a proper clinical and radiological planning has been made. Patients treated with anticoagulant drugs or medically compromised equally can get benefitted by this minimal invasion technique.

** Key words:**Flapless, minimally invasive surgery, dental implant.

## Introduction

Since the 1970s, modern Implantology is based on a concept of surgery with flap elevation. The first incisions followed the protocol designed by Brånemark, performed in the oral vestibule and mucosa, so when flap was replaced, the incision line and suture remained separate from the location of the implant, thus trying to prevent the infection of the surgical area (1,2).

Gradually, several clinical trials demonstrated that a mid-crestal incision gives similar success rates compared to those obtained using the classical protocol. In addition, mid-crestal incision produces less swelling and inflammation. These advantages motived the spread of the use of this type of incisions, being the most commonly used in recent years (3).

However, over the past decade in medicine it has been established the concept of minimally invasive surgery, consisting in taking advantage of advancements experienced in diagnostic techniques and specific surgical instruments, to perform surgical procedures infringing as less damage as possible to the patient through minimal incisions (envelope incision in the beginning, actually without lineal incision (punch)), reducing the size of the instruments. Having managed to reduce considerably the morbidity of these acts and the surgical time, minimally invasive surgery has led to an increase of the degree of patient satisfaction as well. In the field of Oral Surgery, and especially in dental implants surgeries, there was an extended tendency to import this type of minimally invasive techniques. These first steps were led by clinicians, though the scientific evidence arrived years later proving the validity of these surgical approaches.

We must be fully aware of the resorption that crestal bone experiences after surgical procedures involving incision with flap elevation. This occurs unpredictably, as a result of the alteration in the vascularization of the bone periosteum after flap reflection (4). This is also evident after the insertion of dental implants, occurring remodelative processes around the implants, leading to different degrees of crestal bone loss (5,6). Several experimental studies verified that avoid flap reflection on the insertion of dental implants prevents the alteration of the vascularization of the area, improving the behaviour of mucosa, periosteum and periimplantary bone. Atraumatic technique (great respect to the alveolar bone, not exhibition of the bone) provides less crestal bone resorption that could influence on final aesthetic results (7,8).

This together with other advantages of flapless techniques as lower morbidity, (8) better postoperative and the absence of suture, have made it a technique increasingly demanded and used by clinicians in Implantology, both in conventional dental implant surgeries and in implant guided surgery.

The present work aims to produce a thorough review of the literature published on the field of Implantology with flapless surgery (Pubmed, last 15 years), to determine the current scientific evidence of the technique, along with illustrating the results with different clinical cases.

## Advantages of flapless surgery

Many are the advantages that have made flapless surgery of dental implants an act increasingly demanded by clinicians and patients.

• Faster healing of soft tissue: flapless surgery prevents the reflection of soft tissues reducing the surgical trauma. As a result, the necessary process of healing of the wound is minimal, with an absence of scar and its typical complications of conventional surgery as the dehiscence of the flap. The absence of suture in the majority of cases contributes equally to the best postoperative appearance of the surgical area (9).

• Minimal interference on the blood supply: as flapless technique implies only a essential orifice on the mucosa in the flapless technique, blood supply is hardly affected compared to what takes place in surgeries with large flaps which are forced to be designed broad-based in order to avoid flap necrosis (9).

It should be recalled that the vascularization of the underlying bone is determined by three essential sources: major supra-periosteum vessels, vascular plexus of the periodontal ligament, and the vessels of the alveolar bone. With the absence of a tooth, the plexus of the ligament disappears, remaining the vascularization guaranteed due to the two other sources. Under these conditions, the flap reflection entails a loss of the blood supply of the supraperiosteum vessels, so the bone vascularization depends upon its own vessels, which is a poor blood source in the case of cortical bone. This will imply a certain level of bone resorption during healing in cases that occur with a mucoperiosteum flap reflection (10).

Several studies corroborate that bone resorption that follows flap surgery causes a decrease of the vascularzation threatening the final aesthetic results. Thus, Kim in 2009 (7) shows in one study in dogs, than in areas where it was placed a flapless implant presented a much richer vascularization than the area in which the surgery was conventional, thus making a better vascularization of the areas in which flap was not practiced.

Jeong and cols in 2007 (11) published a comparative study in dogs about socket healing after the insertion of an implant with or without flap, showing that sites with flapless technique showed a higher-osseointegration (greater contact bone implant-BIC) and less peri-implantary bone loss, which was measured by greater crestal bone height in these implants. Furthermore, You et al. 2009 (8) repeated the previous model, finding three months after the implant surgery that the flapless technique could reduce gingival inflammation, reduce the height of the junctional epithelium and reduce the bone loss.

Summarizing published studies on flapless surgery, generally showed a broad methodological variability, with an average follow-up of 19 months, bone loss in surgeries without flap that ranged from 0.7 mm to 2.6 mm according to the series, and most of them did not follow a comparative study of flapless surgery parallel to the conventional technique (12). However, in general the flapless surgery showed efficiency and clinical effectiveness, depending on the success of the radiological methods used, the training and the clinical judgment of the surgeon.

As a consequence, there is experimental evidence that in cases without flap reflection the peri-implant mucosa is more vascularized and has reduced dimensions. Also seems to show a lower loss of crestal peri-implant bone, but this is not completely proven yet.

• Reduction of bleeding: one the most advantages of flapless surgery that both the clinician and patient appreciate is the significant reduction in bleeding intra and postoperative. The fact of not reflecting a flap has resulted in much lower blood extravasation and therefore a clean surgical field which provides intervention and shortens your duracion (12).

This feature of minimally invasive surgery makes it especially indicated in elderly patients together with certain pathologies (diabetes, immunodeficiency) in which it is essential to induce the minimum possible damage to the patient and perform the operation in the shortest possible time. On the other hand, and given the current trend of protocols in Haematology that are inclined not suppress anticoagulants and antiplatelet drugs before surgery, the flapless technique is much safer for the treatment of these patients, avoiding the risk of moderate or prolonged bleeding which occur in conventional interventions requiring local haemostatic measures.

• Reduced surgical time: the absence of flap and suturing greatly simplifies the surgery, shortening its duration in most of the cases (12-14). However, we should not forget that this type of surgery requires special concentration when it comes from a technique without direct vision of the bone. For this reason, planning the intervention normally needs a greater dedication and time than conventional implant surgeries (for virtual planning).

In a prospective multicentre study, Becker and cols (14) evaluated the technical flapless placement of implants and determined that, besides being a predictable procedure, it was done in a shorter period of time compared to the conventional technique, with 28 minutes on average of duration of the surgery. However, Lindeboom and van Wijk (15) in a report on the year 2010, did not find significant differences in the duration of the procedure between the two techniques.

• Lower morbidity and an increase on patient comfort: all the studies agree that the postoperative period in these cases is much less symptomatic in contrast to conventional surgery (9,10,12-14). As patients declare a more confortable post-operative, are also much more satisfied with the treatment.

The studies reviewed include the publication of Fortin and cols (16) in 2006 and Nkenke and cols (17) in 2007 which found that flapless technique was associated with a statistically significant reduction of postoperative pain measured by visual analogue scale (VAS) taking on account the duration of pain and analgesic consumption.

• High survival rates: In Brodala’s systematic revision, (12) there are fourteen studies, with a total overall of 2040 implants in 778 patients with 19-months-average monitoring. Its results show a survival of 98.6% in the prospective studies, and of 95.9% in the retrospectives.

In a recent study, Rousseau (18) in 2010 placed 174 implant in 121 patients using flapless technique and compare with a control group to which conventional surgery is performed; They evaluate success, safety and bone changes, finding a 98,3% of success after two years with flapless technic, not having statistically meaningful differences compared to flap technique.

Also, Jeong *et al.* (19) in 2011 conducted a prospective essay over 432 implants achieving 100% of success after a year, with an average bone loss of 0,3 mm. They Conclude that flapless technique is predictable, managing to preserve Crestal bone and peri-implantary mucous health.

## Limitations and complications of flapless technique

As noted from the revision of the scientific evidence, flapless technique presents certain limitations as well which are analyzed below:

• A blind technique.

The lack of flap reflection and the small diameter of mucous openness make a minimal surgery field exist, thus the vision is very limited, being hindered the correct view of cortical, the form of the crest or the concavities. This will ease the arising of complications such as fenestration of cortical, bad implant placing and its bad angulation.

As a consequence of all this, it will be fundamental to make a correct previous diagnosis, both clinical and radiological, as well as a proper surgery planning in order to prevent improvisations and intraoperatory complications (10,18).

In addition to having various radiological researches, specially the ortopantomography and conic beam CT in which the dimensions in the zone to intervene will be seen, it is important to have different clinical resources to determine bone crest width in order to take the decision of whether to conduct or not flapless technic; therefore the use of caliber, endodontic files to evaluate gingival size, crest palpation, etc… will help taking decisions on this kind of cases.

These limitations make this technique, according to various authors, restricted to experienced surgeons that can obviate the limitations it presents. Domínguez Campelo and Domínguez-Cámara carried out in 2002 an essay making flapless technique along 10 years, finding a 25% of complications in the cases treated in the first year, and a decreasing incidence in the following years until achieving the lack of them in the 10th year, what authors blame on the learning curve (10).

• Risk of damaging anatomic structures

The limits on the view have as consequence the possibility of damaging neighbour structures such as cortical, specially the buccal cortical, neighbour teeth roots, important nerves or the sinus. However, even though it is a relatively frequent clinic situation in inexperienced hands, very few essays consider this circumstance (Dominguez Campelo 2002, (10) Cannizzaro 2007, (20) Wittwer 2006 y 2007, (21,22) Di Giacomo (23)). It would be interesting that the prospective series included among their variables the presence of injuries to neighbour structures, relating them to the surgeon’s experience.

• Difficulty of keratinized gum

There is some controversy about the role that plays the around-implants keratinized gum, and the success in the long term of them. While there are authors that have defended that lack of keratinized gum does not influence on the success of implants in the long term, (24-26) the currently most-followed trend is that, although it is not essential, the failure rates are higher when there is little or no keratinized gum around the implant, (27-29) idea that matches with our results.

• Impossibility of flap handling for aesthetic reasons

Not lifting a flap and limiting the openness to just a few millimeters, makes very difficult to conduct this periodontal plastic surgery technics to increase the volume of soft tissues buccal to the implant, or improving the situation and volume of the papilla. These operations get to improve the aesthetic of rehabilitations, ensuring at the same time long-term stability of soft tissues around the implant. For this reason, in those cases in which there is little volume of soft tissues it will be better to conduct a conventional surgery for improving the situation of peri-implantary soft tissues.

• Impossibility of evaluating and treating bone defects 

For the same reasons, low visibility prevents the correct evaluation of bone crest and determining the existence of irregularities such as dehiscences or fenestrations that may compromise the correct intraosseus placing of the implant. Crestal defects go equally undetected and cannot be properly regenerate or regularized.

Therefore, in the event of clinical or radiological bone injuries suspicions, it would be more advisable to proceed with a flap reflection to be able to properly see the surgery area and to apply, if needed, bone regeneration processes which ensure long-term stability of peri-implantary tissues.

## Case Reports

**Figure 1:**
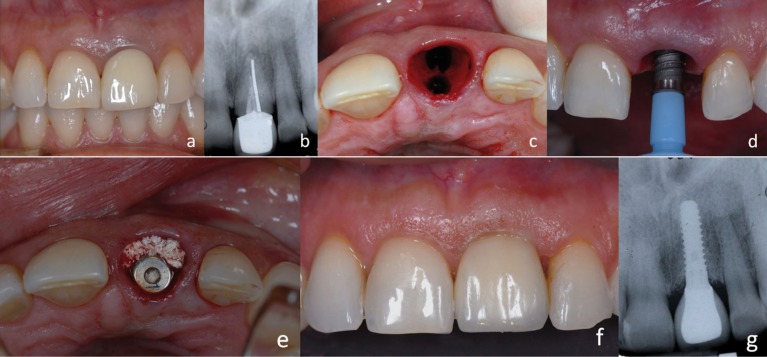
a) Central incisor without future, b) Intraoral radiography, c) Extraction of the tooth, d) Insertion of the immediately implant, e) Regeneration of the coronal gap, f) Frontal view of the prosthetics crown, g) Postoperative x-ray.

From all that has been said so far emerges that flapless technique requires a high technical level, and that it has to be preceded by an exhaustive study on the zone to intervene, with a proper clinical and radiological exploration. Some of the clinical recommendations that stem from the published evidence are showed below along with some clinical cases:

-Case 1:

34-year-old patient, without medical history of interest, suffering a coronary fracture of the right upper central that had gone through root canal treatment 3 years ago and that was carrier of a metal-ceramic crown (Fig. 1). The impossibility of a conservative restoration made consider the convenience of its extraction and reposition by implant-supported crown. The need of preserving as much as possible the aesthetic, especially of soft tissues, made decide to place an immediate implant without flap reflection, following the concepts of minimally invasive surgery.

For that we proceeded with the thorough extraction by ultrasonic technic of the radicular rest and the drilling of the socket in palatal situation to avoid fenestrations, and leaving enough space for proper bone wall formation during the healing and osseointegration process (Fig. 1). Finally a 4 x 12mm Klockner Essential Cone® implant was placed and the resulting gap was filled in by buccal, with deproteinised bovine bone (Bottis Cerabone®) (Fig. 1), to take advantage of its osseoconductive properties and to ease proper bone filling to give correct support to buccal soft tissues.

To ease the adaptation of soft tissues and the upkeep of a proper emergency profile, a temporary acrylic implant-supported crown was placed immediately which was adapted onto a provisional abutment, finishing the adjustment to it with a very polished composite microfilling to avoid plaque retention and mucus inflammation.

Three months after, an impression of the implant and of the emergency profile achieved with the temporary was taken, and a cemented metal-ceramic crown over a specific abutment was made. In the year revision, the aesthetic aspect was healthy and satisfactory, conserving the aesthetic of soft tissues, papillas and height of buccal marginal gum (Fig. 1). Radiographically showed a good bone integration and no signal of periimplantary crestal bone loss.

**Figure 2:**
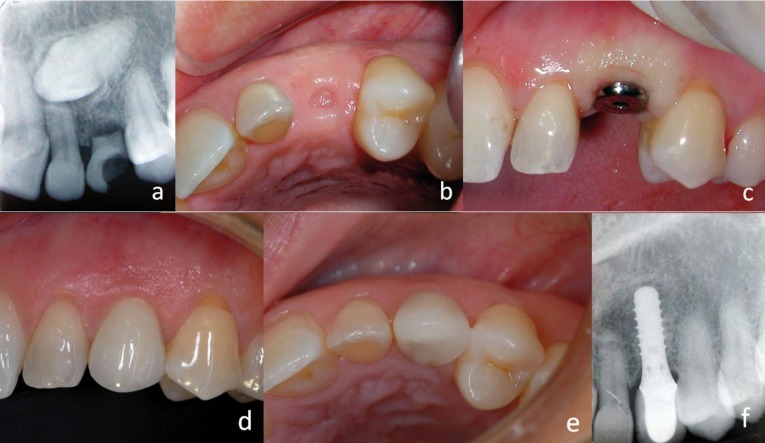
a) Pre-operative X-ray, b) Crestal view of the edentulous space after the extraction of the temporary tooth, c) Placement of the implant, d) Final crown, vestibular view, e) Definitive crown, occlusal view f) Postoperative x-ray.

-Case 2:

41-year-old patient, without medical history of interest, attending due to still presenting a deciduous left upper canine, which shows mobility and crown caries. The definitive canine is impacted in palatal position. The extraction of the impacted tooth and deferred placement of a dental implant for restoring the lost canine was put forward to the patient. (Fig. 2) The extraction was made with the least ostectomy possible, calling for several odontosections to preserve as much bone quantity as possible and to ease the proper bone healing of the zone.

After a waiting period of 5 months, the implant was placed. A thorough radiological clinical exploration promoted the decision of transmucosal implant placement, using flapless technique (Straumann Tissue level implant®, Switzerland), given the good bone availability and the proper maturity of soft tissues (Fig. 2). A 4 mm diameter transmucosal punch was used aiming to make a hole that would adjust to the diameter of the implant’s neck avoiding as much as possible the injure of soft tissues and underlay bone tissue that usually take place when lifting a mucousperiostic flap by the conventional technique. Once the implant was inserted, a healing cap, that occupied the entire hole made, was placed, thus preventing the need of suturing (Fig. 2). After a symptomless postoperative, controlled with a couple of analgesics, two months were waited for impression taking and a cemented metal-ceramic crown was made. 15 days after the definitive restoration was placed, making an appointment for the patient for its periodic revisions.

After 5-year, the crown shows a proper aesthetic aspect with an excellent conservation of periimplantary soft tissues, proper height of papillae and gingival health, preserving a proper thickness of tissue at a buccal cervical level of crown (Fig. 2). Radiologically, after five years, the correct osseointegration can be seen, without evident signs of bone loss, remaining the crestal level at the height of union between the polished neck and the rough surface of the implant (Fig. 2).

**Figure 3:**
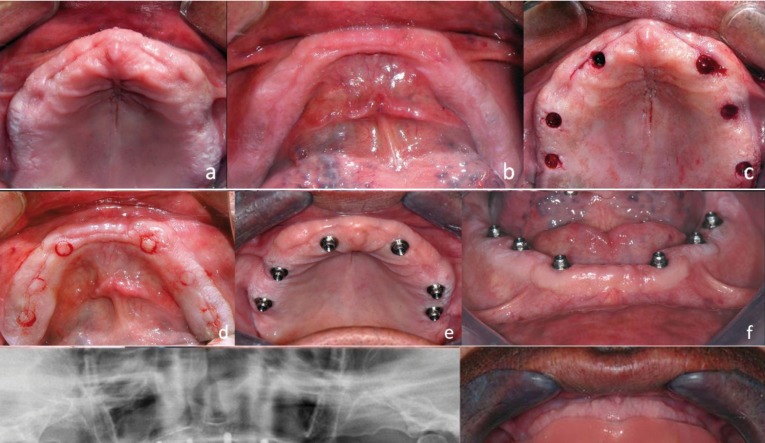
a) Occlusal view of edentulous upper maxilla, b) Occlusal vision of mandible, c) Access to the bone through small mucous fenestrations in the upper jaw, d) Access to the bone through small mucous fenestrations in the mandible, e) Implants placed in the maxilla, f) Implants placed in the mandible, g) Postoperative orthopantomography, h) Upper and lower full denture placed, intraoral view.

-Case3:

70-year-old patient, polymedicated, with a chronic heart disease and undergoing treatment with oral anti-clotting drugs (Dicumarínicos, Sintrom®), who request complete implant-supported rehabilitation by upper and lower hybrid metal-resin prostheses. From the clinical and radiological study emerges that there was good bone availability (Fig. 3); the age and clinical situation of the patient required a minimal-invasion surgery intervention, thus placement of implants by flapless technic is picked up.

Hybrid prostheses technique allows the placing of implants in good bone availability places, not demanding to be subject to so much precision and thoroughness as required in fixed prostheses cases. For this reason it was decided to place six upper and six lower implants, adequately distanced from each other for a proper distribution of occlusal loads. Small circle incisions were made using a 4mm circular scalpel blade and mucous taps were later pulled away by micro periosteal elevators, leaving exposed the underlay bone (Fig. 3).

Afterwards, dental implants were placed using parallelization pins to achieve parallel fixations between them, which will ease the later design of the prostheses, as well as a lingual access to the transocclusal fixation screws of the same. Intervention time was minimum compared to other flap technicques, and patient’s bleeding was equally negligible and easily controllable in spite of not removing the anti-clotting drugs, following the advice from the patient’s hematologist.

Two months after, osseointegration has been achieved adequately and soft tissues presented a good appearance, with keratinized gum around all implants (Fig. 3). Impression taking was carried out and tests begun for the two hybrid prostheses making, its placement and its proper occlusal adjustment. Five years later, the prostheses are still in mouth with a proper function, having the patient an acceptable hygiene. Radiologically it can be appreciated adequate bone levels around the implants, lacking of signs of failure of the osseointegration.

## Conclusions

Flapless technique in Implantology falls within the concept of minimally invasive surgery that has been taking prominence throughout last years in different medical disciplines. In Implantology, this technique allows to make intervention with a minimum aggression to both the bone and soft tissues, shortening the surgery time and achieving high levels of satisfaction by the patient.

However, the technique is not exempt from complications and limitations; the main obstacle of flapless surgery is the fact of limited visibility of the drilling and during implant placement, so the risk of causing wrong bone directions or damaging neighbor structures is higher than with the conventional technique. The impossibility of performing bone regeneration or soft tissues handling technics would be the other great inconvenience of the technique.

For all this, flapless surgeries should be restricted to well-selected cases in which a proper clinical and radiological planning has been made. Patients treated with anticoagulant drugs or medically compromised equally can get benefitted by this minimal invasion technique.
